# The effect of psychological contract on turnover intention among nurses: a meta-analytic review

**DOI:** 10.1186/s12912-023-01496-2

**Published:** 2023-10-05

**Authors:** Xianying Lu, Jing Yang, Dingxi Bai, Mingjin Cai, Wei Wang, Jiali He, Xiaoyan Gong, Chaoming Hou, Jing Gao

**Affiliations:** School of Nursing, Chengdu University of Traditional Chinese Medicine, Chengdu, Sichuan Province China

**Keywords:** Psychological contract, Turnover intention, Nurses, Occupational health, Meta-analysis

## Abstract

**Background:**

The turnover intention (TI) of nurses is common, posing a threat to modern healthcare organizations. Psychological contract (PC) is a predictor of TI, affecting significantly nurse’s TI. However, the extent of the association between PC and nurse’s TI is unknown. We performed a meta-analysis to quantitatively analyze the relationship between PC and nurse’s TI.

**Methods:**

We searched nine electronic databases from inception to July 2023. Observational studies were included using a retrieval strategy related to PC and TI. Meta-analyses of common effect and random effect models were performed using R software with Spearman or Pearson correlation coefficients. Meta-regression, subgroup analysis, publication bias, and sensitivity analysis were also carried out .

**Results:**

Eighteen studies including 8,908 nurses were identified. Based on various PC-related perspectives, 16 studies explored nurses’ TI in terms of the content and three-dimensional structure of PC. Of these, 9 studies reported the negative direction of the correlation between PC and TI (*r* ranged from − 0.20 to -0.45), whereas 7 studies reported the positive direction of the correlation between PC and TI (*r* ranged from 0.32 to 0.50). The PC total and its dimensions were found to have moderately significant associations with TI, with the exception of the PCE and PCE-I. Additional, 2 studies reported the relationship between the outcome of PC and TI, the PCF, PCB, and PCV were powerful predictors of nurses’ TI. Meta-regression and subgroup analysis found that only nurses working in specialized departments might be the source of heterogeneity.

**Conclusions:**

To our knowledge, this was the first meta-analysis to quantitatively examine the relationship between PC and TI among nurses. The findings reaffirmed the necessity for healthcare administrators and the medical profession to valued nurse’ good interpersonal, social support, humanistic environment, and meet nurses’ psychological and spiritual needs in addition to their material demands. Moderators of the connection between PC and TI, based on meta-regression and subgroup analyses, should be carefully explored as they may aid in identifying nurses’ TI. Additional, longitudinal research, as well as mixed research, should be conducted to more comprehensively explore the relationship between PC and TI.

**Supplementary Information:**

The online version contains supplementary material available at 10.1186/s12912-023-01496-2

## Introduction

Turnover intention (TI) is the propensity for employees to leave their current positions and look for new ones [1]; Although it may not always lead to actual turnover, regarded as an essential cognitive process prior to the occurrence of resignation behavior, it is the best reliable antecedent variable to forecast turnover behavior [2]. The stronger the TI, the more likely people will act in a turnover-like manner [3]. Nurses, the largest occupational group in the health sector, have immeasurable value in achieving the goals of universal health coverage and sustainable development [4]. However, the TI and actual turnover of nurses poses a threat to today’s healthcare organizations due to the high-risk nature of nursing, insufficient allocation of human resources, high work-related pressure, low social status, as well as the changing organizations-employee relationships and the unique nature of the nursing profession in the modern day. The turnover rate of nurses in China is reported to be 2.15-5% [5, 6], compared to figures of 10.1–20.4% in South Korea [7], 18.7% in the USA [8], and 12–34% in private hospitals in Indonesia [9]. As is known to all, consistency in nurse-provided care is essential to ensure continuity in care delivery and health quality [10]. Low nurse turnover rates allow for accumulating and expanding nurses’ experience and competence, resulting in better-specialized care. The absence of continuity or stability care is more likely, however, when turnover is excessive, and even if an equal number of new nurses are available to replace lost staff, the lack of clinical experience and solid nursing skills is unlikely to come into play soon, thus exerting great influence on the nursing management. Additionally, it entails both direct costs (such as selection, hiring, and training) and indirect costs (such as emotional instability and lax conduct among other employees) [11], ultimately resulting in weariness and decreased productivity [12]. Therefore, organizations should create effective retention strategies in the event of a nurse shortage by identifying turnover antecedents to take preventative measures to minimize the associated direct and indirect costs.

How to retain nurses at the workplace has thus been a hot issue discussed by domestic and overseas nursing management up to now. According to recent study, low salary, poor work environment, excessive workload, and inconsistency with personal standards all contribute to nurses’ turnover [13, 14], with the most common justification being that their pay was too low [15–17], but Hasselhorn et al.[18] discovered in the study of nurses’ dissatisfaction with salary that nurses typically perceive disparity between what they pay and gain at work, and that this perception is the primary driver of turnover. Similarly, Purvis et al. [19] also noted that the reason nurses leave is not lie in low wages and poor working conditions but rather what they actually require from their employment exchange relationship and whether these needs are met. In addition, Shore [20] found that poorer control management and interrelationships between staff were the key factors contributing to nurses’ willingness to leave. The above-mentioned factor is referred to as psychological contract, leading nurses to have a tendency to leave [21–24].

Psychological contract (PC), as an exchange relationship, is used to comprehend the nature of the working relationships and involves mutual the responsibilities that employers and their employees have to one another [25]. The initial empirical studies focused on corporate employees, librarians, instructors, and knowledge workers. Over time, the notion spread widely into the field of nurse management. In the field of nursing, it refers to nurses’ comprehension and perception about the reciprocal responsibilities that nurses and hospitals must provide for [25], including the nurse’s responsibility to the hospital and the hospital’s responsibility to the nurse. These commitments may include pay for performance, promotion opportunities, and training and level of responsibility provided [26]. PC can arise through overt promises, previous interactions and observations the employee makes. Employees tend to have better work attitudes and conduct properly when they perceive their PC have been fulfilled [27]. Employees, however, may negatively respond in return if the organization fails to fulfill or violates such obligations by adjusting their contributions, displaying undesirable behaviors, or turnover [28, 29]. PC as a psychological bond between nurses and nursing managers is the basis of human resource management, which has a great relationship with the input balance of PC between hospital and nurse and the nurse shortage crisis. As mentioned by Takase, the "true management cost" created by managers’ failure to methodically manage the PC of nurse, resulting in the unfavorable effects of staff burnout, decreased job satisfaction, decreased work commitment, and staff turnover [24]. Furthermore, in hospitals today, there are an increasing number of young, highly educated nurses who care more about the independence and autonomy of values in the employment relationship, have higher career development needs than in the past, pay more attention to the quality of work life, and want to be recognized and respected. Since the PC focuses more on the psychological expectation and demand of nurses, it is necessary to lower nurses’ turnover rates by exploring the perception, expectation, and attitude of nurses towards the hospital-nurse relationship from a new perspective.

Although a substantial amount of empirical research has demonstrated that PC is a strong indicator of an nurse’s TI, the degree of correlation and the criteria for measuring PC varies widely (r ranging from − 0.01 to − 0.484 or 0.078 to 0.541) [30–33]. Meta-analysis fully utilizes the existing research data, combining the outcomes of studies for the same research purpose to expand the sample size and enhance test effectiveness. However, to date, there has not been a quantitative review or meta-analysis on the relationship between PC and TI among nurses. The sole meta-analysis on the correlation between PC and TI included 51 studies published before 2007, and the research subjects were not nurses [29]. Therefore, given the importance of the PC in the workplace, we carried out a systematic review and meta-analysis, with Pearson and Spearman correlation coefficients as the evaluation basis, to compile the available data and more precisely assess the relationship between PC and TI among nurses, thus offering suggestions for focusing on PC, investigating antecedents, and putting strategies in place to lessen nurse’s TI.

## Methods

This systematic review was conducted following the Preferred Reporting Items for Systematic Reviews and Meta-Analyses (PRISMA) guidelines (**Supplementary material, Appendix A**), which was registered on PROSPERO: CRD42023395416. As this was a review with meta-analysis, approval from an ethics committee was not required.

### Search strategy

We conducted eight databases, including PubMed, Embase, Web of Science, Cochrane, China National Knowledge Infrastructure (CNKI), VIP Database for Chinese Technical Periodicals (VIP), Wanfang Data, and Chinese Biomedical (CBM), from the inception up to 5 July 2023. Combinations of MeSH terms, Emtree synonyms, and free words were used in the literature search. The search terms comprised including “nurses/nurse/personnel, nursing/nursing personnel/registered nurses/nurse,registered/nurses, registered/registered nurse” AND “turnover intention/employee turnover/turnover/intention to quit/demission/intent to leave/quit/departure” AND “psychological contract,” were used without date restrictions. In addition, reference lists of the retrieved articles were manually checked to identify additional relevant studies. The specific search strategy is shown in (**Supplementary material, Appendix B**).

### Inclusion and exclusion criteria

The criteria were as follows: (1) the participants were nurses; (2) reporting data on the correlation between PC and TI, including Spearman (*r*_*s*_) or Pearson (*r*) correlation coefficients; and (3) research design was cross-sectional, case-control, or longitudinal design (using baseline data). The exclusion criteria were as follows: (1) studies that were not in English or Chinese language; (2) studies with incomplete data or data that could not be analyzed; (3) duplicate articles and/or data (selected the most recent article).

### Data extraction and outcomes

Two reviewers (LXY and YJ) independently screened the literature and extracted data after documents being imported into Endnote X9. The process of literature screening was as follows: exclude the duplicate studies; read the titles and abstracts to exclude clearly irrelevant articles (unrelated to our outcome of interest) based on the inclusion criteria; and read the full text to to further determine their suitability. The following data were extracted: study characteristics (first author, publication year, country, study design, and type of working department), characteristics of the participants (age (presented as minimum~maximum), gender, sample size, percent of males, percent of permanent nurse, percent of bachelor degree or above, and tools used to measure PC and TI), and effective outcome data (Spearman (*r*_*s*_) or Pearson (*r*) correlation coefficients). Any disagreements in data were resolved by a third party (BDX).

### Quality assessment

Two reviewers (LXY and YJ) independently evaluated the quality of included observational studies using Joanna Briggs Institute (JBI) Checklist for Analytical Cross Sectional Studies [34]. Articles were scored ‘yes’, ‘no’, ‘unclear’, or ‘not applicable’ for the following: (1)criteria for inclusion in the sample clearly defined, (2)study subjects and the setting described in detail, (3)exposure measured in a valid and reliable way, (4)objective,standard criteria used for measurement of the condition, (5)confounding factors identified, (6)strategies to deal with confounding factors stated, (7) outcomes measured in a valid and reliable way, and (8)appropriate statistical analysis used. The item would be scored one if the answer was “yes” Otherwise, it would be scored zero. Any disagreements in data were resolved by a third party (BDX).

### Statistical analysis

The meta-analysis was performed using the “meta” package in R version 4.1.3. Heterogeneity was assessed by the *I*^*2*^ statistic and *Q*-test (*P*-value); if *P* > 0.10 and *I*^*2*^ < 50%, a fixed effects model was chosen; otherwise, a random effects model was adopted. This analysis approach involves converting correlation coefficients into Fisher’s z values, calculating the summary of Fisher’s z, and then converting this to a summary correlation coefficient (*r*) [35]. When heterogeneity occurred, meta regression, subgroup analysis, and sensitivity analysis were performed to assess the source of heterogeneity. Meta-regression analysis was performed to assess the potential effect of important covariates that may lead to heterogeneity. Significant clinical heterogeneity was processed by subgroup analysis or a leave-one-out method by iteratively removing the included study of sensitivity analysis. In this study, meta-regression was performed to explore whether the differences on the type of department (specialist department or others), gender, age, nurse type (permanent nurse or contract worker), and degree of education, when covariates were statistically significant, with *P* ≤ 0.05. Furthermore, we performed a subgroup analysis of those variables and a leave-one-out method by iteratively removing the included study of sensitivity analysis. Then, sensitivity analysis was also used to detect the stability of the results. Meanwhile, funnel plots and Begg’s and Egger’s tests were used to detect publication bias. The correlation coefficient (r) could vary between − 1 and 1; the strength of the effect size between 0 and 0.2 was considered small, between 0.2 and 0.5 was medium, and over 0.5 as large[36]. Before meta-analysis, the Spearman correlation coefficient (*r*_s_) extracted from the literature was converted into the Pearson correlation coefficient (*r*). The conversion formula is as follows [37].

$$r = 2\sin \left( {{r_s},\frac{\pi }{6}} \right)$$

## Result

### Study selection

**Fig. 1:**
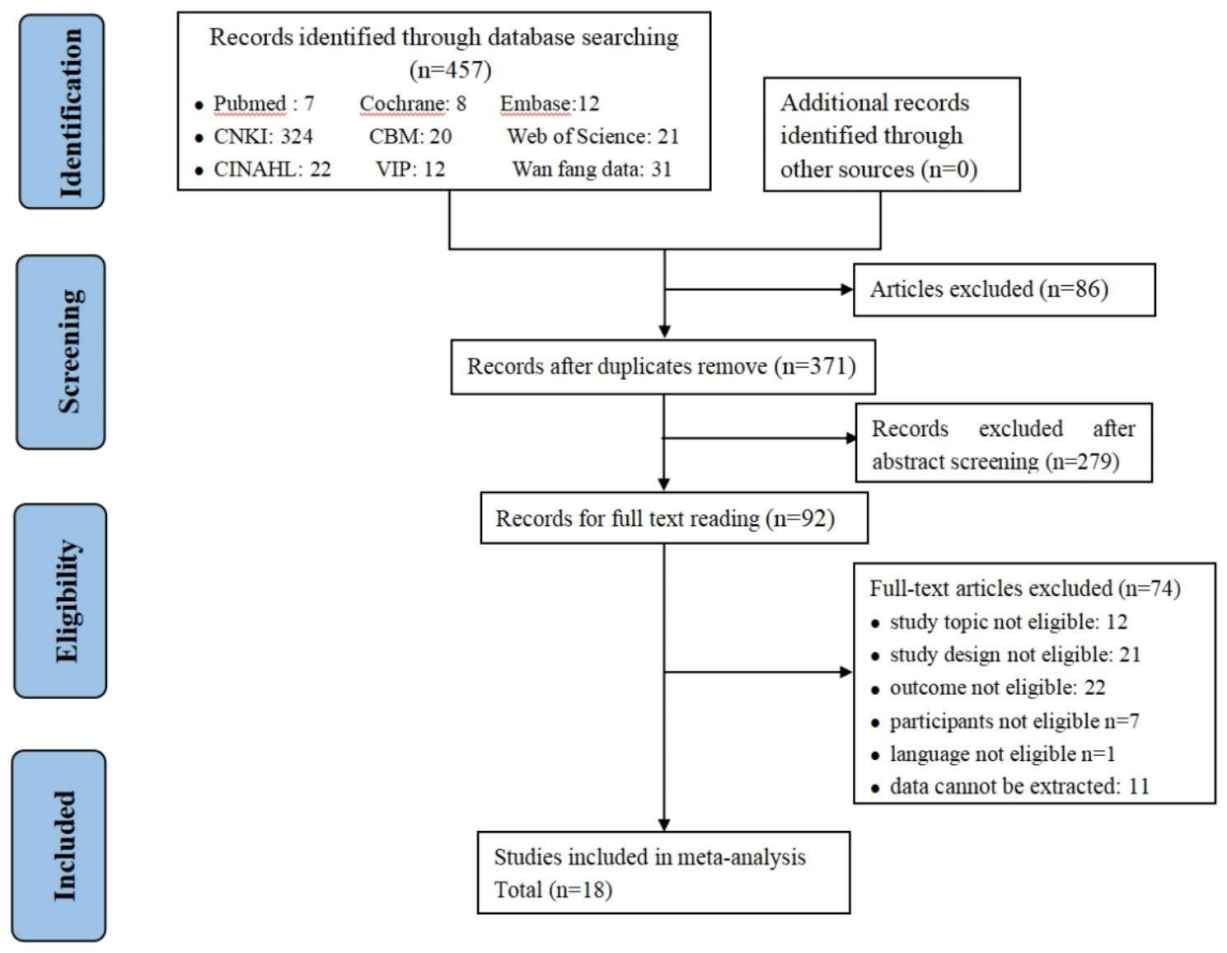
Flow diagram of literature search

The search identified 457 relevant studies from nine databases, 86 studies were excluded due to duplication, and 279 studies were omitted based on titles and abstracts. Of these, 92 studies were selected for full-text screening. After reviewing the full texts, 18 articles [21–24, 30–33, 38–47] met the eligibility criteria. The reasons for exclusion and the process details are given in Figure. 1.

### Study characteristics

A total of 18 studies were conducted across fourteen studies occurred in China, one in Japan, and one in Australia, including 8,908 nurses (ranging from 120 to 2865) of which 4,842 were female, 545 were male and 3,521 nurses did not report gender, primarily working in hospitals and nursing homes. All were cross-section studies. Nine studies used the scale developed by Fu-Rong Chen to assess nurses’ PC. This scale has two sub-scales: organizations responsibility (the responsibility provided by the organization to employee) and the employee responsibility (the responsibility provided by employee to the organization), and each of these two sub-scales has three dimensions: normative responsibility, interpersonal responsibility, and development responsibility. In other words, the more obligations both sides provide for each other (assuming that the hospital provides more responsibilities for nurses), the lower the nurse’s TI will be. Seven studies used the scale created by Fu-Rong Chen, which is made up of two sub-scales: the hospital responsibility (nurses perceive the responsibilities and obligations that hospitals should bear for nurses) and the nurse responsibility (nurses perceive the responsibilities and obligations that nurses should bear for hospitals). The two sub-scales, which are respectively three dimensions, are realistic responsibility, developmental responsibility, and team responsibility. In other words, the nurses’ TI is higher when the two sides perceive that the other side needs to shoulder more responsibility and obligation (assuming that the nurse perceives that the hospital should shoulder more responsibility and obligation for the nurse). Details are shown **in** Table 1. Measuring the quality of studies by JBI, all studies were judged as having a low risk of bias (**Supplementary material, Appendix C**).

**Table 1:** The characteristics of 18 studies included in this meta-analysis

Study (first author, year, country)	Specialist department	Sample size (female/male)	Age (min~max)	Age ≤ 30 (%)	Permanent nurse (%)	Bachelor degree or above (%)	PC measure	TI measure	R (minimum value ~ maximum value) (the number of r were included in this review, including PC total and its dimensions and TI )	R type	Quality
Yang T T^1^, 2017 (China)	NA	285 (285/0)	21~30	100	5.96	22.46	Li Y	Pei Y	-0.019 ~ -0.192 (9 r were included in this review )	Pearson	8
Li N, 2016 (China)	paediatric	358 (285/0)	20~55	NA	34.64	36.59	Li Y	Li DR	-0.231~-0.484 (7 r were included in this review )	Pearson	8
Liu X X, 2013 (China)	nursing homes	202 (NA/NA)	NA	6.93	70.79	13.36	Li Y	Li DR	-0.178 ~ -0.371 (7 r were included in this review )	Pearson	8
Yang T T^2^, 2016 (China)	NA	583 (568/15)	≤ 30	97.43	8.23	35.68	Li Y	Pei Y	-0.084 ~ -0.301 (9 r were included in this review )	Pearson	8
Yu J, 2016 (China)	emergency	120 (118/2)	21~52	NA	NA	19.17	Li Y	Li DR	-0.229 ~ -0.474 (7 r were included in this review )	Pearson	6
Zhou J, 2018 (China)	ICU	221 (211/10)	NA	76.92	12.22	57.47	Chen FR	Li DR	0.078 ~ 0.475 (9 r were included in this review )	Pearson	8
Guo X R, 2016 (China)	Mixed	180 (NA/NA)	20~49	76.11	0	56.67	Chen FR	Wang YZ	0.352 ~ 0.532 (8 r_s_ were included in this review )	Spearman	8
Wu J, 2016 (China)	neurosurgery	128 (128/0)	19~54	NA	41.41	30.47	Li Y	Li DR	-0.235 ~ -0.476 (7 r were included in this review )	Pearson	8
Li Y X, 2016 (China)	nursing homes	1046 (873/173)	19~62	NA	NA	0	Li Y	Li DR	-0.232 ~ -0.465 (7 r were included in this review )	Pearson	8
Jin C Z, 2016 (China)	Mixed	150 (NA/NA)	20~55	18.67	0	60	Chen FR	Wang YZ	0.428 ~ 0.541 (8 r were included in this review )	Pearson	8
Chen F R, 2009 (China)	Mixed	402 (402/0)	20~59	38.81	NA	23.63	Chen FR	FARH JL	0.348 ~ 0.522 (8 r_s_ were included in this review )	Spearman	7
Wang J, 2022 (China)	NA	448 (354/94)	NA	3.13	NA	NA	Li Y	Li DR	-0.191 ~ -0.418 (7 r were included in this review )	Pearson	8
Chen N, 2015 (China)	oncology	270 (268/2)	22~48	NA	65.56	71.85	Chen FR	self-designed	0.342 ~ 0.452 (6 r were included in this review )	Pearson	6
Guo L, 2010 (China)	Mixed	2865 (NA/NA)	NA	46.18	NA	NA	Chen FR	Wang YZ	-0.01 ~ 0.18 (6 r were included in this review )	Pearson	8
He X C, 2023 (China)	psychiatry	285 (182/103)	NA	NA	56.84	74.04	Chen FR	Pei Y	0.35 ~ 0.472 (7 r were included in this review )	Pearson	8
Feng Y, 2022 (China)	paediatric	142 (NA/NA)	≥ 20	5.6	4.23	21.13	Li Y	Li DR	-0.621 ~ -0.701 (3 r were included in this review )	Pearson	6
M Takase, 2016 (Japan)	Mixed	766 (701/65)	22~62	50.65	NA	NA	Maertz and Boyar	Maertz and Boyar	-0.54	Pearson	8
J Rodwell, 2016 (Australian)	Mixed	457 (376/81)	25~44	NA	NA	NA	Robinson and Morrison	Chatman and Wayne	-0.16 ~ 0.51 (4 r were included in this review )	Pearson	8

Abbreviations: NA = not avaliable. Measure tools: PC = Psychological Contract, TI = Turnover Intention, Li Y = questionnaire designed by Li Yuan (2006), Chen FR = questionnaire designed by Chen Fu-Rong (2008), Pei Y = questionnaire designed by Pei Yan (2007), Li DR = questionnaire designed by Li Dong-Rong (2000), Wang YZ = questionnaire designed by Wang Yu-Zhi (2005), FARH JL = questionnaire designed by FARH JL (1998), self-designed = self-designed questionnaire. r = the result of Pearson test, r_s_ = the result of Spearman test

### Meta-analysis

This study explored the relationship between PC and TI among nurses from various PC-related perspectives. The first examined the PC’s three-dimensional structure and content, while the second focused on the PC’s outcomes (including psychological contract fulfillment (PCF), psychological contract promises (PCP), psychological contract breach (PCB), and psychological contract violation (PCV)).

### Correlation between PC’s content and dimensions and TI

16 studies from China explored nurses’ TI in terms of the content and three-dimensional structure of PC [25, 48]. The 16 studies that were included, however, showed two diametrically opposed orientations of the connection between PC and TI due to the specificity of the scale for measuring PC. That is, the PC scale compiled by Yuan Li showed that nurses’ TI decreases as the hospital provides her more duties and responsibilities, whereas the PC scale compiled by Fu-Rong Chen demonstrated that nurse’s TI increases when she perceives the hospital should bear more responsibilities and obligations for her. The total r coefficient of these 16 studies was therefore meaningless in determining whether the nurses’ PC is positively or negatively correlated with TI; instead, the correlation between PC and TI was conducted directly based on the trends of scale for measuring PC, as shown **in** Table 2. Among these, 9 studies reported the negative tendency for the correlation between PC and TI, and the PC scale was developed by Yuan Li. The pooled *r* coefficient between PCT and TI was − 0.43 (95% confidence interval (CI) = -0.53 to -0.33), having moderate negative correlations, with substantial heterogeneity (*I*^*2*^ = 83.9%, *P* < 0.01); the strength of this effect size just like the relationships associate PCO and its dimensions, PCE-N, and PCE-D with TI, ranged from − 0.20 to -0.45. The value zero was not in the 95% CI, except for the employee responsibility dimensions, suggesting all correlations were negative and statistically significant. 7 studies reported the positive direction of the correlation between PC and TI, and the PC scale was developed by Fu-Rong Chen, The pooled *r* coefficient between PCT and TI was − 0.49 (95% CI: -0.40–-0.58; I^2^ = 0%, P = 0.61), the result demonstrated that PCT and all its dimensions were moderate positively correlated with TI (r were from 0.32 to 0.50), and all correlations were statistically significant.

**Table 2:** The summary correlation between PC total score and its dimensions and TI

The variables	N	Sample size	Heterogeneity			Model	Summary r (95%CI)
			I^2^(%)	Q	P		
The psychological contract scale developed by Yuan Li (2006)							
Psychological contract total score (PCT)	9	3312	83.9	49.75	<0.01	random	-0.43(-0.53,-0.33)
Organizations responsibility dimensions (PCO)	3	1010	95.7	46.74	<0.01	random	-0.45(-0.86,-0.04)
Normative responsibility (PCO-N)	8	3170	45.1	12.76	0.08	common	-0.33(-0.39,-0.28)
Interpersonal responsibility (PCO-I)	8	3170	83.2	41.65	<0.01	random	-0.41(-0.51,-0.32)
Development responsibility (PCO-D)	8	3170	77.2	30.69	<0.01	random	-0.38(-0.46,-0.30)
Employee responsibility dimensions (PCE)	3	1010	97.1	68.58	<0.01	random	**-0.33(-0.83,0.17)**
Normative responsibility (PCE-N)	8	3170	42.5	12.17	0.09	common	-0.20(-0.25,-0.15)
Interpersonal responsibility (PCE-I)	8	3170	75.6	28.69	<0.01	random	-0.18(-0.26,-0.10)
Development responsibility (PCE-D)	8	3170	61.8	18.33	0.01	random	-0.22(-0.28,-0.16)
The psychological contract scale developed by Fu-Rong Chen (2008)							
Psychological contract total score (PCT)	2	506	0	0.27	0.61	common	0.49(0.40,0.58)
Hospital responsibility dimensions (PCH)	4	953	0	1.87	0.6	common	0.50(0.44,0.57)
Realistic responsibility (PCH-R)	7	4373	95.7	140.08	<0.01	random	0.36(0.24,0.48)
Developmental responsibility (PCH-D)	7	4373	94.4	106.64	<0.01	random	0.41(0.29,0.52)
Team responsibility (PCH-T)	7	4373	97.0	199.31	<0.01	random	0.41(0.27,0.56)
Nurse responsibility dimensions (PCN)	4	953	88.8	26.71	<0.01	random	0.49(0.30,0.69)
Realistic responsibility (PCN-R)	7	4373	92.9	84.14	<0.01	random	0.32(0.20,0.44)
Developmental responsibility (PCN-D)	7	4373	97.2	213.60	<0.01	random	0.37(0.21,0.52)
Team responsibility (PCN-T)	7	4373	93.1	86.46	<0.01	random	0.39(0.28,0.51)

### Correlation between the PC’s outcome and TI

Additionally, literature from Australia and Japan by Takase and Rodwell discussed the connection between the PC’s outcome and TI. Large, significant associations between PCF (r = -0.54, 95% CI = -0.67 to -0.41), PCB (r = 0.56, 95% CI = 0.47 to 0.65), and PCV (r = 0.52, 95% CI = 0.43 to 0.62) and TI were discovered (Table 3), but since only one or two studies were included, we should proceed with caution in drawing this conclusion.

**Table 3:** The summary correlation between the outcome of PC and TI

The variables	N	Sample size	Heterogeneity			Model	Summary r (95%CI)
			I^2^(%)	Q	P		
Psychological contract fulfillment (PCF)	2	1223	79.8	4.95	<0.01	random	-0.54(-0.67,-0.41)
Psychological contract promises (PCP)	1	457	——	——	——	——	-0.16(-0.25,-0.07)
Psychological contract breach (PCB)	1	457	——	——	——	——	0.56(0.47,0.65)
Psychological contract violation (PCV)	1	457	——	——	——	——	0.52(0.43,0.62)

### Meta-regression and subgroup analysis of the association between PC total score and TI

High heterogeneity was found in the results of the meta-analysis using only the scales developed by Yuan Li (*I*^*2*^ = 83.9%, *P* < 0.01); meta regression, subgroup analysis, and sensitivity analysis were carried out to determine the reason for the heterogeneity. The sample size, publication year, specialist department and percentage of males, age ≤ 30, permanent nurse, and bachelor’s degree or above were chosen as the covariate. Meta-regression analysis showed that specialist department (Estimate = 0.1045; Z = 2.5408; P < 0.05; 95% CI [0.0238 to 0.1848]) and percent of age ≤ 30 (B = 0.3099; Z = 2.1454; P < 0.05; 95% CI [0.0268 to 0.5930]) positively impacted the effect size of PC and TI relationship. According to these results, when the percentage of specialist departments and age ≤ 30 increases, the effect size heterogeneity of the PC and TI relationship also increases (Table 4). The subgroup analysis findings revealed that gender was not a significant moderator for this relationship; however, the effect size of this relationship among the specialist department was significant. Nurses from specialty departments such as pediatric, emergency, ICU, or nursing homes had a higher correlation between PC and TI (Table 5).

**Table 4:** Meta-regression analysis of the association between PC total score and TI

Variable	Estimate	SE	Z	P	95% CI	
					L	U
Sample size	0.0001	0.0002	0.4425	0.6581	0.0003	0.0005
Publication year	-0.0213	0.0213	-1.0812	0.2796	-0.0649	0.0188
Specialist department	0.1043	0.0410	2.5408	**0.011**	0.0238	0.1848
Percent of males	-0.3957	0.6328	-0.6252	0.5318	-1.6359	0.8446
Percent of age ≤ 30	0.3099	0.1444	2.1454	**0.0319**	0.0268	0.5930
Percent of permanent nurse	-0.0920	0.2242	-0.4105	0.6815	-0.5313	0.3473
Percent of bachelor degree or above	0.6492	0.5969	1.0877	0.2767	-0.5207	1.8191

Note P^a^ value for the between-subgroup difference; P^b^ value for the heterogeneity within subgroups by Q test.

**Table 5:** Subgroup analysis of the association between PC total score and TI

Moderators	N	Sample size	Summary r (95%CI)	P^a^	Heterogeneity		
					I^2^(%)	Q	P^b^
Specialist department							
Yes	6	1996	-0.50(-0.57,-0.42)	0.025	52.2	10.47	0.06
Unclear	3	1316	-0.29(-0.45, -0.13)		86.8	15.17	<0.01
Gender							
Female	2	703	-0.37(-0.56,-0.18)	0.9116	73.6	3.79	<0.01
Mix	4	2137	-0.39(-0.54,-0.23)		88.3	25.58	<0.01

Note P^a^ value for the between-subgroup difference; P^b^ value for the heterogeneity within subgroups by Q test.

### Sensitivity analysis and publication bias

Sensitivity analysis was conducted, and the findings remained constant. when each study was excluded serially (**Supplementary material, Appendix D**). It indicated that the results of this meta-analysis were stable. Funnel plot together with Egger tests were not conducted to estimate the publication bias since the number of included studies was less than 10.

## Discussion

### Summary of key findings

To the best of our knowledge, this was the first systematic review and meta-analysis to quantitatively investigate the association between PC and TI in nurses using correlation coefficient (r). Interestingly, only two studies from Australia and Japan explored the outcomes of PC (PCF, PCP, PCB, and PCB) among the included 18 studies, the remaining 16 studies from China explored nurses’ TI in terms of the content and three-dimensional structure of PC [48], which reflected the specific differences in diverse employment relationships in different countries, ethnicities and cultural contexts [49]. The JBI score ranged from 6 to 8 points, indicating the high quality of the included studies. Our results suggested that the effect size of PC and most of its dimensions on TI was medium in the health industry, and PC is an important indicator of TI among nurses, consistent with previous research.

### Correlation between PC’s content and dimensions and TI

The PC Scale, developed by Yuan Li in 2006 or by Fu-Rong Chen in 2008, is the most common scale adapted from the Rousseau Psychological Contract Survey Tool in China; consisting of two subscales of organizational responsibility (also named hospital responsibility) and employee responsibility (also named nurse responsibility) [50, 51]. For Yuan Li’s scale each scale included three dimensions of normative responsibility, interpersonal responsibility, and development responsibility. Furthermore, Fu-Rong Chen’s scale entailed three dimensions: realistic responsibility, development responsibility, and team responsibility. Among them, Yuan Li’s normative responsibility and interpersonal responsibility are basically the same as the contents of Fu-Rong Chen’s realistic responsibility and team responsibility, respectively.

The results of this study showed that PC, measured by Yuan Li or Fu-Rong Chen, was moderately correlated with TI, and a stronger association was found between organizational responsibility and TI compared with employee responsibility, indicating that nurses paid more attention to the perception of organizational responsibility fulfillment. Among them, in the organizational responsibility subscale, both measured by Yuan Li and Fu-Rong Chen demonstrated that interpersonal responsibility (also named team responsibility) showed a stronger correlation with TI, compared with results of the developmental responsibility and normative responsibility (also named realistic responsibility), which was consistent with the results of Liu Xiaoxia and Chen Furong et al [39, 51]. Although most studies generally agreed that low income and poor working conditions were the main reasons for nurses’ TI, this is indisputable. Our study also explained the reason for the nurses’ TI from a different aspect, which was organizational interpersonal responsibility. In the employment relationship between nurses and hospitals, nurses value good interpersonal and social support within the hospital and a humanistic environment where they are valued and respected more than the material and financial rewards provided by the hospital. Our findings also support the idea that nurses’ TI was not primarily influenced by their perceptions of low material rewards and poor working environments [19]. Accordingly, for hospital management, flexible shift arrangements based on clinical needs and nurse’s personal characteristics, frequent affirmation of nurses’ work performance, emphasis on nurses’ mental health, and emphasis on recognition, mutual respect, coordination and cooperation, as well as humanistic care are of great significance to reduce nurses’ TI.

While in the employee responsibility dimensions (also name nurse responsibility dimensions), PCE-D and PCN-T exhibited the highest correlation with TI. Nurses’ developmental responsibility is the responsibility and obligation that nurses must undertake for the long-term development of hospitals and nursing majors [50]; nurses’ team responsibility is the responsibility and obligation that nurses assume for the team building of the hospital [51], both are essential to the long-term growth of the hospital and the nursing profession. This result further proved the unique characteristics of nurses’ PC compared with general enterprise employees, that is, whether the hospital could genuinely respect, recognize and care for the nursing team, display humanistic care for nurses and provide social and psychological support which directly affect the nurses’ willingness to leave, and further in turn affect their behavior at work.

### Correlation between the PC’s outcome and TI

Additionally, PCF, PCB, and PCV were significant indications of TI among nurses based on the results of PC. Our research demonstrated that PCB was positively connected with TI and that it can identify employees’ TI, which is consistent with the previous study by Zhao et al.[29]. But the connection between PCB and TI in our study (r = 0.56) was larger than Zhao et al (r = 0.42), publication year and study population may be one explanation for this result. the literature we included was published before 2023 and concentrated on the group of nurses, whereas the study by Zhao et al. included the literature prior to 2007 and did not place constraints on the study population. Today, employees are more eager for career development. They focus more on the independence of their employment relationships and the autonomy of their values, and want to be recognized and appreciated, whereas PC meets employees’ psychological needs and expectations, which is why our study shows a greater correlation. However, due to the small number of included studies (n = 1), it is crucial to handle the findings of these analyses with caution.

### Meta-regression and subgroup analysis

Meta-regression and subgroup analysis showed that only nurses in specialized departments, such as pediatric, neurosurgery, emergency, ICU, and nursing homes, might be the source of heterogeneity, and the correlation between PC and TI was higher than that in other departments. The high incidence of nurse turnover in hospitals’ specialty departments resulted from the special service objects of patients, their complicated and changing conditions of patients, many critical illnesses, and the department’s unique characteristics. While in nursing homes, there is lacking a perfect management system, the salary and remuneration do not entirely commensurate with the amount of labor, nursing staff in nursing institutions do not have a perfect career promotion route, and the recognition of nursing staff in nursing homes and society is not high, resulting in a higher willingness of nursing staff in nursing homes to leave. Therefore, for hospital administrators, the TI of nurses should be reduced from the perspective of PC according to the unique characteristics of each specialized department. Moreover, the subgroup analysis for country, gender, degree of education, age, and nurse type (permanent nurse or contract worker) was not performed due to the limited sample size. Therefore, the sample size should be considered when specifying appropriate measures in future studies.

### Theoretical and practical implications

The shortage of nurses is a long-standing and persistent problem faced by health care system worldwide, greatly restricting the development of the nursing profession. However, replenishing the nursing workforce involves complex issues at multiple levels and cannot be achieved overnight. Therefore, scientific management of nursing human resources is essential under the existing objective conditions[52]. As a practical human resource management theory, PC has been introduced into companies and is highly valued and used to manage the business-employee employment relationship. The introduction of PC, an important theory of management psychology, into nursing management is also of great importance. This study has demonstrated the relationship between PC and TI among nurses. Learning the relationship between PC and TI can provide an up-to-date theoretical background for managing nurses’ TI. Most importantly, this study adds up to the knowledge of nurses and healthcare managers about PC and TI.

Furthermore, the literature on the connection between PC and TI is scant, and there has not been a meta-analysis study looking at this association in the health sector. Our findings filled this gap. Additionally, our meta-analysis study could compare various industries using the results of prior research; hence, other researchers could compare their findings to those of our study.

### Limitations and future research

This study had some potential limitations. First, cross-sectional data can only demonstrate an association rather than offer causality. Second, a social desirability effect may have affected nurses’ PC and TI. Third, nurses’ TI at the time before and during the epidemic may have different effects. These may also aid in preventing method biases [52–54]. Fourth, the review only included studies from China, Japan, and Australia, but lacking studies from other countries like the United States, Europe, and Africa. All studies that examined nurses’ TI in terms of the content and dimensions of PC were from China due to the various PC-related viewpoints, whereas these studies that discussed nurses’ TI in terms of the outcome of PC originated from Japan and Australia. This may be related to certain differences in employment relations under different national, ethnic and cultural background [49], which was also the biggest but unavoidable problem at present. Our investigation revealed three potential avenues for future study. First, current research on nurses’ PC and TI is sparse relative to research on the PC among other professionals. Future research should use experimental or longitudinal designs to gain strong causal insight. Second, since PC and TI are subjective psychological states, qualitative and quantitative research should be done to examine nurses’ perceptions and experiences of PC and TI to learn more about which interventions effectively reduce nurses’ TI. Third, a larger sample comprising multiple hospitals should be added to the study to validate the conclusions of this study among different countries based on the various PC-related viewpoints.

## Conclusion

In conclusion, based on the various PC-related perspectives, 16 studies explored nurses’ TI in terms of the content and three-dimensional structure of PC, among these, 9 studies reported the negative direction of the correlation between PC and TI (*r* ranged from − 0.20 to -0.45), while 7 studies reported the positive direction of the correlation between PC and TI (*r* ranged from 0.32 to 0.50), except for the PCE and PCE-I, moderate, significant relationships were found between PC content and its dimensions and TI, and a stronger association was found between organizational responsibility and TI compared with employee responsibility. In the organizational responsibility subscale, the relationship between PCO-I (also named PCH-T ) and TI was a stronger correlation, in the employee responsibility subscale, PCE-D and PCN-T shown the highest correlation with TI, indicated that in the employment relationship between nurses and hospitals, nurses valued good interpersonal, social support, and a humanistic environment where they are valued and respected more than the material and financial rewards provided by the hospital. Meta-regression and subgroup analysis found that only nurses in specialized departments might be the source of heterogeneity, and the correlation between PC and TI was higher than in other departments. Additionally, 2 studies reported the relationship between the outcome of PC and TI, the PCF, PCB, and PCV were powerful predictors of nurses’ TI. Therefore, on the one hand, in addition to offering material assistance to nurses, healthcare managers and the medical community should be mindful of their psychological and spiritual needs directly. Most significantly, this study raises the awareness of PC and TI among nurses and healthcare managers.

### Electronic Supplementary Material

Below is the link to the electronic supplementary material

Supplementary Material 1

## Data Availability

All the data are available from the corresponding author up on a reasonable request.
